# Association between Handwashing Behavior and Infectious Diseases among Low-Income Community Children in Urban New Delhi, India: A Cross-Sectional Study

**DOI:** 10.3390/ijerph182312535

**Published:** 2021-11-28

**Authors:** Khalid M. Khan, Rishika Chakraborty, Stephen Brown, Rasheda Sultana, Alec Colon, Devinder Toor, Pooja Upreti, Banalata Sen

**Affiliations:** 1 Department of Population Health, College of Health Sciences, Sam Houston State University, Huntsville, TX 77341, USA; sbrown@shsu.edu (S.B.); mxs218@shsu.edu (R.S.); 2 Department of Environmental and Occupational Health, School of Public Health, Indiana University Bloomington, Bloomington, IN 47405, USA; rchakra@iu.edu; 3 Massachusetts General Hospital Cancer Center, Boston, MA 02114, USA; acolon344@gmail.com; 4 Amity Institute of Virology and Immunology, Amity University Uttar Pradesh, Noida 201313, India; dtoor@amity.edu (D.T.); poojauprety232@gmail.com (P.U.); 5 Sustainable Environment and Ecological Development Society (SEEDS), New Delhi 110022, India; bonosen@gmail.com

**Keywords:** diarrhea, handwashing critical time points, respiratory infections, schoolchildren

## Abstract

Diarrheal diseases and respiratory infections (RI) are two leading causes of childhood mortality in low and middle-income countries. Effective handwashing at critical time-points may mitigate these diseases. However, there is a lack of published data investigating this association in school-aged children in India. This study is part of a larger prospective handwashing intervention study in a low-income community in New Delhi, India examining the associations between handwashing behavior and diarrhea and RI in schoolchildren. This current study reports the findings of the baseline survey administered to 272 mother–child dyads. Children aged 8–12 years, and their mothers, were recruited from six schools. A baseline questionnaire was used to collect sociodemographic data, handwash behavior, and mother-reported recent diarrhea and RI incidence among the children. Handwashing before and after preparing food, after defecation, and after cleaning dishes significantly reduced the odds of diarrhea by over 70%, and of RI by over 56%. Using a clean cloth after handwashing lowered odds of diarrhea and RI by 72% and 63% respectively. Around 60% of the participants believed that handwashing could prevent diarrhea and RI in their children. There was a low prevalence of handwashing at critical time-points and a poor perception regarding handwashing benefits. To improve handwashing behavior, hygiene promotion programs need to understand what motivates and hinders handwashing in vulnerable populations.

## 1. Introduction

Diarrhea and respiratory infections (RI) continue to be leading causes of childhood morbidity and mortality in developing countries [1]. Diarrheal diseases and RI account for about 1.3 and 2.6 million annual deaths respectively [2]. However, the burden of these diseases is disproportionately higher in low and middle-income countries (LMICs), including India [3]. The World Health Organization (WHO) has estimated that diarrhea and RI account for approximately 30% and 18% respectively, of the global burden of disability adjusted life years for the people of India [4]. There are multiple etiological agents for infectious diarrhea and RI. Diarrhea is commonly caused by bacteria such as *Escherichia coli* and *Vibrio cholerae* [5]; by viruses such as rotavirus and adenovirus and parasites such as *Giardia* [6]. The predominant bacterial and viral agents causing RI include *Streptococcus pneumoniae, Klebsiella pneumoniae*, rhinoviruses and influenza viruses [7,8,9].

Children are most vulnerable to these infectious diseases when they lack access to safe water and sanitation, have poor hygiene behavior in and outside their homes, and experience nutritional deficiencies [10]. Children attending elementary school are especially vulnerable because these diseases also impede academic and physical development, leading to impaired cognitive performance [11] and stunting growth [12]. Disruptions during childhood development often lead to poor quality of life in adulthood [13,14].

Improved hygiene practices are considered among the most cost-effective social interventions for reducing the burden of diarrheal diseases and RI. Handwashing is the most prominent and affordable hygiene practice for low-income populations [15,16,17]. Handwashing with soap can reduce the risk of diarrhea by 47% [18] and the risk of RI by 24% [19]. Despite extensive governmental and non-governmental public health educational efforts to promote hygiene practices, the rate of compliance is still critically low in LMICs, including in India. Globally, only 19% of the population wash hands after fecal contact [20] while according to one study, only 2% handwash in India after fecal contact [21]. Because of poor handwashing behavior, incidence of infectious diseases remains high in India, costing an estimated USD 23 billion every year [22]. By targeting sensitive groups in low-income populations, particularly children, public health practitioners can not only reduce morbidity and mortality from RI and diarrhea, but also improve overall economic wellbeing.

Community or school-based handwashing interventions have been shown to raise hygiene awareness, lower risk of infectious diseases, and reduce school absenteeism in students [23,24,25]. These children, in turn, can become influential in encouraging family members to adopt better hygiene practices [26]. Most published handwashing hygiene interventions have targeted children younger than 5 years of age [27,28,29]. However, risk for fecal contact is higher for children in school. Older children demonstrate high frequency hand-to-mouth transfer as they come into contact with fecal matter while playing on the school grounds and sharing crowded washrooms that often lack proper handwashing facilities. Consequently, school children are at high risk of developing diarrheal diseases and RI [30]. Although several studies have reported the impact of poor handwashing on infectious diseases among children in India, the protective effects of hygiene practices (i.e., behavior and timing of handwash at home or school) has not been explored. Understanding this may be important because handwashing behavior of the mothers and children could reduce diarrhea and RI in children [31,32]. In this study, we examined the associations between handwashing behavior and diarrhea and respiratory infections in children between 8 to 12 years of age. We present the findings of a cross-sectional study using the baseline data from a prospective educational intervention on school children, living in low-income urban slum communities in East Delhi, India.

## 2. Materials and Methods

### 2.1. Study Design and Setting

This current study utilizes the baseline data of a prospective handwashing behavior change intervention study conducted in East Delhi, which began in August 2018 and is currently ongoing. The intervention study is a collaborative project involving the faculty, staff, and students of Indiana University Bloomington (IUB) in the United States and three Indian institutions, Ambedkar University Delhi (AUD), Amity University Uttar Pradesh (AUUP) and Sustainable Environment and Ecological Development Society (SEEDS).

SEEDS has been working with several public (government-funded) elementary schools (locally known as primary schools) located in low-income communities in East Delhi. Most of the children attending these schools belong to migrant families who relocated from rural communities across India to East Delhi for work. From a list of 50 primary schools located in East Delhi, six schools were randomly selected for the hand washing behavior change intervention project. Mother–child dyads were selected from these six schools. The present study reports the findings of baseline surveys administered to the mother–child dyads before the start of the behavior change intervention. Since this study reports results from only the baseline data of the project, we present our findings for all participants from the six schools together. Ethical approval for this study was obtained from the Institutional Review Boards at both IUB and AUUP.

### 2.2. Sample Size and Participant Recruitment

Third to sixth grade teachers, from the six selected schools, identified eligible child participants based on our inclusion criteria. To be included, child participants had to be between 8–12 years of age, be students attending third to fifth grades, have no physical or mental disability, live in urban slums, and have a physically active mother living in the same house with the child. If more than one child in a household was eligible, only one was selected. From an initial list of 482 participants from the same number of households who met the inclusion criteria, we randomly selected 50 potential child participants from each school and reached out to their mothers for informed verbal consent. Although our initial target was to recruit fifty children per school thus achieving a sample size of 300 for the study, 28 participants were unable, or unwilling, to take part in the study, giving a response rate of 90.7%. Among these 28 excluded participants, mothers of 20 were not available when our field staff reached out to them since they moved to another city at the time of recruitment, whereas 8 children were found to have chronic illness not allowing them to attend schools regularly. Therefore, our final sample included 272 randomly recruited mother–child dyads from six schools. We obtained verbal informed consent from mothers and child assent before initiating any data collection activity.

### 2.3. Questionnaire Survey

The Principal Investigator from IUB, USA and the local Co-Investigators trained the SEEDS research assistants (RAs) to conduct face-to-face interviews with the mother–child dyads. The RAs used a structured questionnaire to collect data from the mother–child dyads regarding socio-demographic characteristics, hand-washing practices, beliefs, behaviors, and diarrheal disease and RI incidence among children. Each mother responded to all the questions in presence of the child participant, via a face-to-face interview with our field staff. Standing height of all children was assessed using a measuring tape to the nearest cm (Stanley 33-725 25-Feet FatMax Tape, Stanley Tools, New Britain, CT, USA) and child weight was measured to the nearest kilogram using a calibrated digital weighing scale (Hoffen HO-18 Digital Electronic Weighing Scale, ACE Incorporation, India). The height and weight measurements were used to compute body mass index (BMI).

### 2.4. Handwash Behaviors and Practices

Mothers and children self-reported their usual handwashing time-points, behaviors, and facilities at home and in school. Each handwashing behavior and occasion was recorded as binary variables with “yes” or “no” answers. For example, “used liquid soap to wash hands” and “washed hands before eating” both had answer options “yes” or “no”.

### 2.5. Health Outcomes

The two child health outcomes assessed in this study were diarrhea and RI in the past 12 months, as reported by the mothers. Diarrhea was measured by the question, “Did your child get any diarrheal diseases in the past 12 months?” and RI with “Did your child get any respiratory illnesses in the past 12 months?” with “yes” or “no” answer options. The mothers were also asked to elaborate what type of diarrhea or RI the child suffered in the past 12 months. Diarrhea was defined as passage of 3 or more loose or liquid stools per day or more frequently than normal for the individual [33]. RI was defined as pneumonia, cough, fever, chest pain and shortness of breath, cold, inflammation of the airways or any other symptoms of respiratory illnesses [34].

### 2.6. Statistical Analysis

Statistical analysis was performed using R statistical programming software v3.6.0. Sociodemographic characteristics of the mother–child dyads are presented as mean ± standard deviation for continuous variables and number (%) for categorical variables. Bivariate analyses were performed using Chi-square tests or Fisher’s tests. Separate unadjusted and adjusted logistic regression models were performed to investigate the association of each major explanatory variable such as individual handwashing behavior and critical handwashing time point with the health outcomes of diarrheal and RI incidence. Along with a major explanatory variable, we also considered child age, BMI, mother’s education, and monthly household income as potential covariates in each model. Therefore, five explanatory variables were used in each model. To detect potential multicollinearity in our regression models, we calculated the variance inflation factor (VIF) for each model. We found VIF was less than 1.5 for all models, indicating low correlation among our predictor variables. We defined statistical significance as *p* < 0.05 tests for the hypotheses were two-sided.

## 3. Results

### 3.1. Sociodemographic Characteristics

The sociodemographic characteristics of all child participants are described in Table 1. On average, children were 9 years old, and their mothers were 32 years old. Average child BMI was nearly 15 kg/m^2^, and was within normal range according to WHO reference growth charts [35]. Over half (58.8%) of the child participants were male. About half (53.7%) of the maternal participants had received no schooling, while nearly 70% of the fathers had received some education (primary and/or secondary and above). Three-fifths (60%) of the mothers were unemployed while nearly all (97.2%) of the fathers had some form of employment. Two-thirds (67%) of the households had five or fewer household members, and majority (80%) rented their homes. About 11% of the households had a monthly income of less than or equal to 6000 INR. Diarrhea incidence in the past year was reported in 23.5% of the child participants while RI incidence in the past year was reported in 37.5% of the child participants. Diarrhea and RI incidence was similar between girls and boys (*p* > 0.05).

### 3.2. Handwashing Behaviors and Infectious Disease Outcomes

Handwashing behavior, and its association with diarrheal disease and RI outcomes in children, determined by multivariate logistic regression models, are outlined in Table 2 and Table 3 respectively. Handwashing was practiced by both mother and children in 80.4% of the households. Use of bar or liquid soap was not associated with both of our infectious disease outcomes. As far as handwashing facilities were concerned, having water in a tub with a mug outside the house significantly increased the odds of getting diarrheal disease by 3.14 times. Such unexpected results were not observed for RI. Wiping hands with a clean cloth was significantly protective against both infectious disease outcomes. The strongest barrier to handwashing was forgetting to wash hands at critical periods, reported by 12.9% of the participants. This significantly increased the odds of getting diarrhea by 3.91 times. Forgetting to wash hands was also associated with higher odds of RI in the unadjusted analyses, but was attenuated after adjusting for covariates. Most handwashing behaviors were not significantly different between girls and boys. However, more girls (89.3%) reported using a bar soap than boys (78.1%); while 23.2% of the girls identified “being tired of handwashing” as a potential barrier, as opposed to only 13% of the boys (*p* < 0.05). The frequency of handwashing behavior by participants who reported incidence of diarrheal disease ranged from 1.6% to 85.9%, across a wide range of behaviors described in Table 2. For those who reported RI incidence, frequency of handwashing behavior ranged from 1.9% to 86.3% for the different behaviors (Table 3).

### 3.3. Timing of Handwash Activities and Infectious Disease Outcomes

Handwash occasions at home and in school, before and after critical time-points, and thier association with diarrheal disease and RI outcomes in children, determined by multivariate logistic regression models, are reported in Table 4 and Table 5 respectively. The prevalence of handwashing at key occasions at home is shown in Supplementary Figure S1. Handwashing before preparing and after preparing food (practiced by ~24% and ~20% respectively) significantly reduced the odds of both diarrheal disease and RI by 50% to 70%. Handwashing after defecation was also significantly protective against diarrhea and RI. However, only 34.2% of the children said they washed hands after defecation. Handwashing after cleaning dishes by hand (practiced by 16.5%) was associated with 85% and 61% lowered odds of diarrhea and RI respectively. At school, handwashing after playing reduced the odds of diarrhea by 57% and of RI by 74%. Nearly 20% of the children reported they washed hands after playing in school. Washing hands after using the toilet increased RI incidence. In addition, handwashing prevalence after eating at home or in school was significantly higher in girls than boys (*p* < 0.01 for both). Around 90% of the girls said they washed hands after eating both at home and in school while 84% of the boys said they washed hands after eating at home and only 64% of the boys reported handwashing after eating in school. For participants who reported incidence of diarrheal disease, frequency of handwashing at critical time points ranged from 4.7%% to 88% across the range of occasions described in Table 4. For those who reported RI incidence, frequency of handwashing at critical time points ranged from 6.9% to 89.2% for the different occasions (Table 5).

### 3.4. Timing of Handwash Activities and Infectious Disease Outcomes

**Figure 1:**
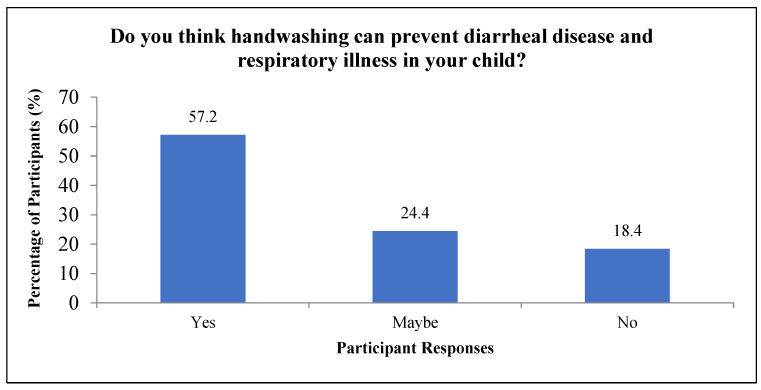
Participant responses for perception of diarrheal and respiratory illness prevention from handwashing.

The mothers’ beliefs regarding the benefits of handwashing are shown in Figure 1. A little more than half of the participating mothers (i.e., 57.2%) believed that handwashing would prevent diarrhea and RI in their children. Almost a quarter of the mothers (24.4%) interviewed were skeptical about the benefits of handwashing, while 18.4% did not believe that handwashing was effective against diarrhea and RI.

## 4. Discussion

We report an association between improved handwashing behavior at critical time-points and a reduction of the mother-reported incidence of diarrheal disease and RI in children. Our results are consistent with several other studies conducted elsewhere, which have identified regular hand hygiene practice as an effective barrier against these two diseases [15,16,23,36]. We have reported some critical time-points of handwashing that are linked to reduced odds of both diarrhea and RI such as before and after food preparations, after defecation, and after dish cleaning by hands. We observe similar findings in other parts of the world among people with low socioeconomic status. Studies conducted in Bangladesh and Ethiopia also found that washing hands before food preparation and after defecation as most important for prevention of diarrhea in children in urban slums [37,38]. Additionally, we found a low prevalence of handwashing at these critical time-points. Similar to our findings, another study in India [21] reported less than 30% of mothers and schoolchildren were handwashing with soap on key occasions, both at baseline and at follow-up.

Even though handwashing with soap is the recommended practice, we found that using a bar soap or a liquid soap was not associated with diarrheal or RI incidence. Similar to our results, two prior studies reported that handwashing with a bar soap was not associated with influenza [39,40] and diarrhea [40]. This might be due to inadequate frequency of handwashing observed in our sample [39]. We identified the use of a clean cloth to dry hands post-handwashing to be significantly protective against diarrheal disease and RI. This is consistent with current handwashing guidelines that recommend using a clean towel to dry hands [41]. Unexpectedly, RI incidence increased when children reported handwashing after using the toilet in school. This may be explained due to inadequate soap use or crowded school toilets leading to improper handwashing practice. Respiratory illness incidence in Delhi is also high due to severe air pollution throughout the year, which may have biased our results.

As expected, we found that forgetting to wash hands was a statistically significant risk factor of diarrhea. This finding is similar to a study in Colombia [42], where forgetting to wash hands was the most commonly reported barrier of personal hygiene promotion. A tub of water with a mug outside the house is a common handwash station in low-income communities in India. Nevertheless, it represents an unhygienic facility that is vulnerable to contamination. We also found that such an infrastructure was significantly associated with increased odds of diarrheal disease incidence, but not with RI. Contrary to our finding, presence of a handwashing station with both soap and water showed no association with diarrhea prevalence, but increased RI prevalence in Kenya, which may have occurred due to unmeasured confounding [40].

Just over half of our study participants (57.2%) believed that handwashing would help in reducing diarrheal diseases or RI. The poor perception and low confidence for the benefits of handwashing among adult females underscores the need for handwashing awareness and intervention programs targeting mothers and children from low-income households in India. A study in Pakistan found that a majority of adult respondents believed overeating to be the most important risk factor of diarrhea. While contaminated food and water were also believed to be causal factors; the participants referred to flies or visible dirt, rather than microscopic bacteria and germs, as the major risk factors for hand contamination and infection transmission [43]. Similarly, a study in Bangladesh reported only a small percentage of the informants believed that handwashing was helpful in reducing RI. The majority believed that keeping a safe distance from the infected individual could be the most effective strategy to prevent RI. In addition, the participants did not think it was always feasible to wash hands after coughing or sneezing [44]. All of these findings indicate that cultural belief regarding the appropriate timing of handwash remains a barrier of sustainable hand hygiene promotion. Future studies can also investigate if mother’s handwashing beliefs impact their children’s attitude towards handwashing and handwashing practices.

There were some potential limitations in our study. Most results are based on parent-reported data and no direct observation of handwashing behavior was included in the study design. This may have led to recall bias and over-reporting of handwashing due to social desirability bias. Incidence of diarrheal and RI infection was also parent-reported. We acknowledge that our outcome data may also be subject to recall bias. There may be over-reporting, under-reporting and misclassification of diarrheal disease and RI incidence in our sample. However, we asked detailed questions in order to capture the most accurate answers regarding these experiences. It is plausible that the mothers may have had difficulty in remembering the frequency of our outcomes in the past year for their children. To address some of this concern, our outcome was analysed as a dichotomous variable with a “yes” or “no” response to any incidence of the outcome in the past year.

Our study does not report any microbiological data identifying potential pathogens that may have caused diarrhea and RI in the children. Microorganisms cause the most severe outbreaks of diarrhea and RI. Living in crowded areas with poor hygiene and sanitation conditions facilitate easy transmission of microbes. Co-infection with more than one microorganism is also common, causing more serious cases of diarrhea and RI [6]. In our future studies we will incorporate microbial data analysed from samples collected from households and children’s hands. Because this is a cross-sectional study, temporality cannot be established. However, it is well known that poor hand hygiene leads to diarrheal disease and RI. In addition, this study represents handwash behavior in a small subset of the Indian population. Whether the findings are generalizable across other rural and urban populations in India and other LMICs remain unclear. Additionally, there may be confounding by variables that were not measured in this study.

## 5. Conclusions

In our study, we have reported that safe handwashing practices at critical times can reduce the parent-reported risk of diarrhea and RI. Despite being a low-cost prevention measure, we found low prevalence of handwashing at important time-points, and only around 60% of participants believed in its effectiveness. Forgetfulness was observed to be the biggest barrier to handwashing. These study findings will inform our ongoing handwash behavioral change intervention. Based on our results, we recommend future studies focus on understanding what facilitates handwashing such as having an accessible handwashing station, sufficient clean water supply and easy availability of soap while addressing the hindrances, such as forgetfulness and cultural beliefs, with appropriate cues and education to promote improved handwashing behavior by vulnerable mother and child populations.

## Figures and Tables

**Table 1:** Sociodemographic characteristics of the participants (*N* = 272).

Characteristic	Child Participants
	(*N* = 272)
	(Mean ± S.D)
Child’s Age	9.08 ± 1.17
Mother’s Age	31.81 ± 4.45
Child’s BMI	14.99 ± 2.99
	n (%)
Sex	
Female	112 (41.2)
Male	160 (58.8)
Educational status of mother	
No school	146 (53.7)
Primary	93 (34.2)
Secondary and above	33 (12.1)
Educational status of father	
No school	81 (29.8)
Primary	94 (34.6)
Secondary and above	97 (35.6)
Employment status of mother	
Employed	106 (39.0)
Unemployed	166 (61.0)
Employment status of father	
Employed	264 (97.1)
Unemployed	8 (2.9)
No. of people in a household	
≤5	183 (67.3)
>5	89 (32.7)
Monthly household income	
(INR)	
<2000–6000	29 (10.7)
6001–10,000	119 (43.7)
>10,000	124 (45.6)
Home ownership	
Own home	52 (19.1)
Rental home	220 (80.9)
Diarrheal disease incidence	64 (23.5)
Respiratory illness incidence	102 (37.5)

**Table 2:** Unadjusted and adjusted odds ratios for association between handwashing behaviors at home and diarrheal disease outcome.

Behaviors	Diarrhea Incidence*N* = 64	No Diarrhea Incidence *N* = 207	*p* Value ^#^	OR for Diarrheal Disease	95% CI	*p* Value	OR ^a^ for Diarrheal Disease	95% CI	*p* Value
*Soap type*									
Liquid	8 (12.5)	46 (22.2)	0.09	0.50	0.21, 1.07	0.09	0.44	0.17, 1.02	0.07
Bar	55 (85.9)	170 (82.1)	0.48	1.33	0.63, 3.09	0.48	1.69	0.75, 4.20	0.22
*Handwashing facility*									
Basin and tap inside toilet	5 (7.8)	39 (18.8)	0.04	0.37	0.12, 0.89	0.04	0.39	0.13, 0.99	0.07
Basin and tap outside toilet	13 (20.3)	74 (35.7)	0.02	0.46	0.23, 0.87	0.03	0.48	0.23, 0.95	0.04
Water in tub with mug inside	33 (51.6)	105 (50.7)	0.91	1.03	0.59, 1.82	0.91	1.03	0.57, 1.85	0.93
Water in tub with mug outside	16 (25.0)	20 (9.7)	0.002	3.12	1.49, 6.46	<0.01	3.14	1.43, 6.86	<0.01
*Hand drying method*									
Clean cloth	9 (14.1)	89 (42.9)	<0.001	0.22	0.09, 0.44	<0.001	0.28	0.12, 0.59	0.001
Multi-purpose rag	45 (70.3)	97 (46.9)	0.001	2.68	1.49, 4.99	<0.01	1.82	0.96, 3.55	0.07
Air dry	3 (4.7)	27 (13.0)	0.06	0.33	0.08, 0.97	0.08	0.48	0.11, 1.52	0.26
Wipe on clothes	10 (15.6)	48 (23.2)	0.20	0.61	0.28, 1.25	0.20	0.72	0.31, 1.51	0.40
*Barriers to handwashing*									
Too busy	50 (78.1)	177 (85.5)	0.16	0.61	0.30, 1.26	0.16	0.69	0.34, 1.50	0.34
No soap	1 (1.6)	2 (0.9)	0.56	1.63	0.07, 17.26	0.69	1.18	0.05, 13.35	0.89
Too tired	13 (20.3)	34 (16.4)	0.47	1.29	0.62, 2.59	0.47	0.89	0.40, 1.88	0.77
Forgot	18 (28.1)	17 (8.2)	<0.001	4.35	2.08, 9.17	<0.001	3.91	1.75, 8.83	<0.001

^#^ Results from Chi-square test/Fisher test, ^a^ adjusted for child age, BMI, mothers education, monthly household income.

**Table 3:** Unadjusted and adjusted odds ratios for association between handwashing behaviors at home and respiratory illness outcome.

Behaviors	Respiratory Illness Incidence *N* = 102	No Respiratory Illness *N* = 168	*p* Value ^#^	OR for Respiratory Illness	95% CI	*p* Value	OR ^a^ for Respiratory Illness	95% CI	*p* Value
*Soap type*									
Liquid	16 (15.7)	38 (22.6)	0.17	0.63	0.33, 1.19	0.17	0.64	0.32, 1.26	0.21
Bar	88 (86.3)	137 (81.5)	0.31	1.42	0.73, 2.89	0.31	1.71	0.84, 3.65	0.15
*Handwashing facility*									
Basin and tap inside toilet	18 (17.6)	26 (15.5)	0.64	1.17	0.60, 2.25	0.64	1.40	0.69, 2.79	0.34
Basin and tap outside toilet	33 (32.4)	54 (32.1)	0.97	1.01	0.59, 1.70	0.97	1.06	0.60, 1.85	0.84
Water in tub with mug inside	49 (48.0)	88 (52.3)	0.49	0.84	0.51, 1.37	0.49	0.85	0.51, 1.43	0.55
Water in tub with mug outside	15 (14.7)	21 (12.5)	0.61	1.21	0.58, 2.45	0.61	1.04	0.49, 2.20	0.91
*Hand drying method*									
Clean cloth	21 (20.6)	77 (45.8)	<0.001	0.31	0.17, 0.53	<0.001	0.37	0.19, 0.67	0.001
Multi-purpose rag	64 (62.7)	77 (45.8)	<0.01	1.99	1.21, 3.31	<0.01	1.71	0.98, 2.99	0.06
Air dry	14 (13.7)	16 (9.5)	0.29	1.51	0.70, 3.25	0.29	1.60	0.68, 3.76	0.28
Wipe on clothes	18 (17.6)	40 (23.8)	0.23	0.69	0.36, 1.26	0.24	0.75	0.38, 1.41	0.38
*Barriers to handwashing*									
Too busy	77 (75.5)	150 (89.3)	0.002	0.37	0.19, 0.71	< 0.01	0.42	0.21, 0.82	0.01
No soap	2 (1.9)	1 (0.6)	0.55	3.34	0.32, 72.41	0.33	2.05	0.19, 45.55	0.57
Too tired	19 (18.6)	28 (16.7)	0.68	1.14	0.59, 2.17	0.68	0.95	0.48, 1.85	0.88
Forgot	19 (18.6)	16 (9.5)	0.03	2.16	1.06, 4.47	0.04	1.93	0.89, 4.22	0.09

^#^ Results from Chi-square test/Fisher test, ^a^ adjusted for child age, BMI, mothers education, monthly household income.

**Table 4:** Unadjusted and adjusted odds ratios for association between handwashing at critical time points and diarrheal disease outcome.

Critical Time Points	Diarrhea Incidence *N* = 64	No Diarrhea Incidence *N* = 207	*p* Value ^#^	OR for Diarrheal Diseases	95% CI	*p* Value	OR ^a^ for Diarrheal Diseases	95% CI	*p* Value
*Timing of handwash at home*									
Before eating	53 (82.8)	186 (89.9)	0.13	0.54	0.25, 1.23	0.13	0.56	0.25, 1.32	0.17
After eating	53 (82.8)	188 (90.8)	0.07	0.49	0.22, 1.12	0.08	0.74	0.31, 1.87	0.52
Before preparing food	6 (9.4)	59 (28.5)	0.002	0.26	0.09, 0.59	<0.01	0.27	0.09, 0.63	<0.01
After preparing food	3 (4.7)	49 (23.7)	<0.001	0.16	0.04, 0.45	<0.01	0.19	0.04, 0.56	<0.01
After cleaning dishes	3 (4.7)	42 (20.3)	0.002	0.19	0.05, 0.56	<0.01	0.15	0.03, 0.47	<0.01
Cleaning house	5 (7.8)	24 (11.6)	0.39	0.65	0.21, 1.64	0.40	0.56	0.16, 1.56	0.29
After coming from school	8 (12.5)	35 (16.9)	0.40	0.70	0.29, 1.53	0.40	0.68	0.26, 1.61	0.41
After defecating	4 (6.3)	89 (42.9)	<0.001	0.08	0.03, 0.22	<0.001	0.11	0.03, 0.29	<0.001
After urinating	56 (88.0)	191 (92.3)	0.24	0.59	0.24, 1.51	0.25	0.67	0.27, 1.78	0.40
Touching something unclean	5 (7.8)	43 (20.8)	0.02	0.32	0.11, 0.79	0.03	0.26	0.08, 0.68	0.01
*Timing of handwash at school*									
Before eating	47 (73.4)	165 (79.7)	0.29	0.70	0.37, 1.37	0.29	0.73	0.36, 1.51	0.39
After eating	44 (68.8)	158 (76.3)	0.22	0.68	0.37, 1.28	0.22	0.83	0.42, 1.66	0.58
After playing	8 (12.5)	45 (21.7)	0.10	0.51	0.21, 1.10	0.11	0.43	0.17, 0.97	0.06
Touching something unclean	3 (4.7)	25 (12.1)	0.10	0.36	0.08, 1.07	0.10	0.32	0.07, 1.01	0.08
After using the toilet	44 (68.8)	157 (75.8)	0.26	0.70	0.38, 1.31	0.26	0.77	0.40, 1.52	0.44

^#^ Results from Chi-square test/Fisher test, ^a^ adjusted for child age, BMI, mothers education, monthly household income.

**Table 5:** Unadjusted and adjusted odds ratios for association between handwashing at critical time points and respiratory illness outcome.

**Critical Time Points**	**Respiratory Illness Incidence** ***N* = 102**	**No Respiratory Illness** ***N* = 168**	***p* Value ^#^**	**OR for Respiratory Illness**	**95% CI**	***p* Value**	**OR ^a^ for Respiratory Illness**	**95% CI**	***p* Value**
*Timing of handwash at home*									
Before eating	87 (85.3)	151 (89.8)	0.26	0.65	0.31, 1.39	0.26	0.81	0.37, 1.78	0.59
After eating	87 (85.3)	153 (91.1)	0.14	0.57	0.26, 1.22	0.15	0.76	0.33, 1.77	0.52
Before preparing food	12 (11.8)	52 (30.9)	<0.001	0.30	0.14, 0.57	<0.001	0.29	0.14, 0.58	<0.001
After preparing food	12 (11.8)	40 (23.8)	0.01	0.43	0.20, 0.84	0.02	0.44	0.20, 0.91	0.03
After cleaning dishes	11 (10.8)	34 (20.2)	0.04	0.48	0.22, 0.96	0.05	0.39	0.17, 0.82	0.02
Cleaning house	7 (6.9)	22 (14.3)	0.11	0.49	0.19, 1.14	0.11	0.44	0.16, 1.10	0.09
After coming from school	14 (13.7)	28 (16.7)	0.52	0.79	0.39, 1.57	0.52	0.90	0.42, 1.86	0.78
After defecating	13 (12.7)	79 (47.0)	<0.001	0.16	0.08, 0.31	<0.001	0.15	0.07, 0.30	<0.001
After urinating	91 (89.2)	155 (92.3)	0.40	0.69	0.30, 1.64	0.40	0.81	0.34, 1.98	0.64
Touching something unclean	15 (14.7)	33 (19.6)	0.30	0.71	0.35, 1.35	0.31	0.59	0.28, 1.17	0.14
*Timing of handwash at school*									
Before eating	82 (80.4)	129 (76.8)	0.49	1.24	0.68, 2.30	0.49	1.53	0.80, 3.02	0.21
After eating	71 (69.6)	131 (77.9)	0.12	0.65	0.37, 1.13	0.13	0.66	0.36, 1.21	0.18
After playing	9 (8.8)	44 (26.2)	<0.001	0.27	0.12, 0.56	<0.001	0.26	0.11, 0.54	<0.001
Touching something unclean	12 (11.8)	16 (9.5)	0.56	1.27	0.56, 2.78	0.56	1.27	0.55, 2.88	0.57
After using the toilet	83 (81.4)	117 (69.6)	0.03	1.90	1.06, 3.52	0.03	2.46	1.32, 4.80	<0.01

^#^ Results from Chi-square test, ^a^ adjusted for child age, BMI, mothers education, monthly household income.

## Data Availability

The dataset analysed during the current study are available from the corresponding author on reasonable request.

## Supplementary Materials

The following is available online at https://www.mdpi.com/article/10.3390/ijerph182312535/s1. Figure S1. Prevalence of self-reported handwashing at different time points at home.
