# Supplementary material for: Association between Handwashing Behavior and Infectious Diseases among Low-Income Community Children in Urban New Delhi, India: A Cross-Sectional Study
Source: Int J Environ Res Public Health. 2021 Nov 28;18(23):12535. doi: 10.3390/ijerph182312535 (PMC8657428; doi:10.3390/ijerph182312535)
Supplement: Supplementary file 1 [file ijerph-18-12535-s001.zip › ijerph-1425579-supplementary.pdf]

Supplementary Figure:

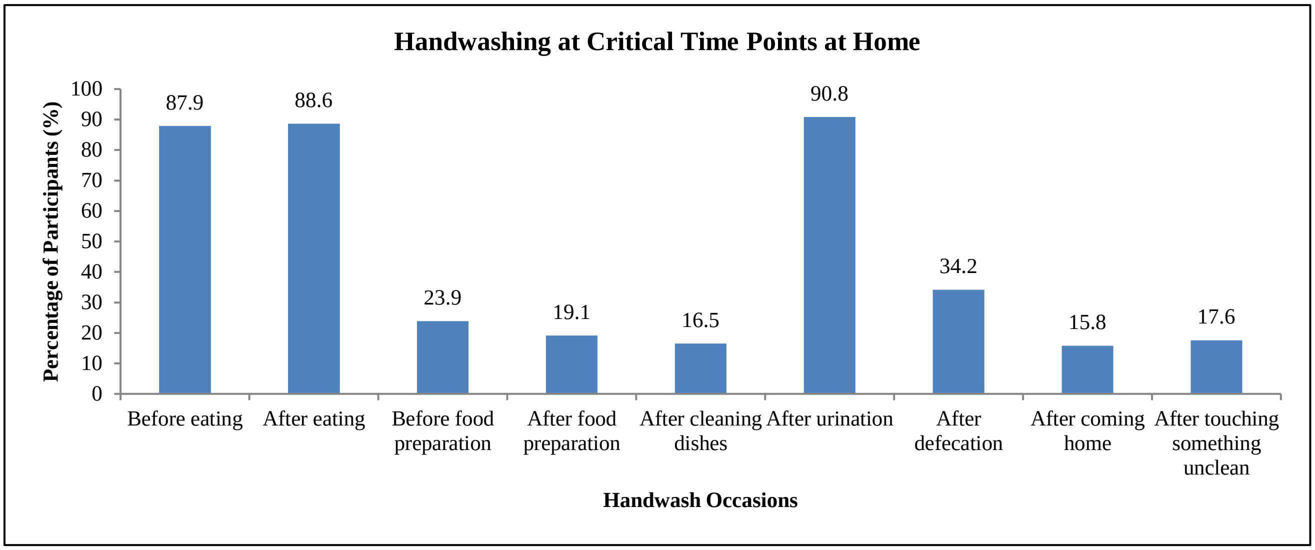

Figure S1. Prevalence of self-reported handwashing at different time points at home
